# Review of the Book *Millennium Development Goals (MDGs) in Retrospect. Africa´s Development Beyond 2015*

**DOI:** 10.1007/s11482-016-9490-7

**Published:** 2016-11-22

**Authors:** Alejandro Migueltorena

**Affiliations:** Instituto Geografía, Historia y Ciencias Sociales. Facultad de Ciencias Humanas, Universidad Nacional del Centro de la Provincia de Buenos Aires, Tandil, Buenos Aires Argentina

Andrews, N. (et al.) (Eds.) Brandon, Manitoba, Canadá. Editorial Board. ISBN: 978-3-319-16165-5. 322 p.

In 2000, leaders from around the world met at the Millennium Summit in New York, Headquarters of the United Nations, in order to channel the different nations towards the path of development. As a result of this meeting a statement, the Millennium Development Goals (MDG) arises and includes among its main goals: reducing extreme poverty and hunger, fighting infant and maternal mortality, stopping the spread of diseases (mainly HIV and malaria) and promoting universal access to primary education, reducing gender gap and articulating economic growth with environmental sustainability. 2015 was the deadline to achieve those objectives, thus, making this book a need to evaluate and reflect on the progress made in the African continent regarding these stated objectives. Therefore, through twenty chapters, a detailed study of the different policies made and implemented in the continent is developed, including obstacles encountered in different concrete experiences, the particularities relating to each of the regions, the influence of developed international context and the challenges presented in the African scenario after 2015.

The authors also discuss the notion of development, indicating that, in general, schools of thought that have influenced the development of programs and policies related to this subject come from intellectuals or institutions regarding European and North American origin, them being outside the particularities of the African continent. This has aroused criticism since the simple extrapolation of recipes of thought from the first world in order to achieve development does not take into account the diversity of cultures that inhabit the continent and their respective worldviews. Another important aspect is the analysis of the variables and indicators in development programs, which are based on a purely quantitative approach to measure the well-being of communities; this overstates the importance of economic growth over qualitative aspects, which are usually relevant to local populations.

As regards the structure of the book, it consists of an introduction, five parts in which it delves into some of the aspects of the policies aimed at fulfilling the Millennium Development Goals and a conclusion. The introduction gives an idea about the general context that crosses the African continent at the time of the agreed deadline for achieving the targets, indicating both aspects related to variables such as international conflicts and local crises that affect the result of the policies. A methodological discussion on the variables and indicators used to measure development also aroused. Then, in the first part, the implementation of programs aimed at reducing poverty and including social sectors in marginal condition is assessed. Throughout three chapters, various projects implemented in different parts of Africa that have achieved mixed results in meeting the goals are discussed. In this sense, the main causes that hindered the implementation of these policies were analyzed: regional inequalities, unequal income distribution, high unemployment and informality rates (which particularly affect women and young people), lack of provision of basic services, political instability and armed conflicts, economies vulnerable to international commodity prices, lack of confidence in the institutions, and especially, the matter of a failed state.

The second part is focused on the study of access to education and its strengthening. The papers state that the objectives of development programs have focused on the need to enroll children in primary school but this is not enough to advance towards development if the quality of education is poor. The need to establish a relationship between primary and higher education is stressed, improving teacher training to increase educational levels. The need to include the theoretical and conceptual framework of social capital and entrepreneurship in the post-2015 scenario is also mentioned because it can help poor communities to participate in the reforming of education funding as well as in policy design.

The third part is aimed at addressing gender issues, stressing the need to rethink the cultural patterns that prevent poor rural women (the most vulnerable social sector of society) from accessing the land, resources and services, as this would mitigate the negative effects of climate change and improve the living conditions of a significant proportion of the population. A directly proportional relationship is established between the reduction of the gender gap and the potential for economic development.

Subsequently, the fourth part is devoted to studying the issues of population health. For this reason, the importance of acquiring programs on sexual and reproductive health in the most vulnerable regions is analyzed; the importance of identifying cultural practices that hinder the successful implementation of policies aimed at improving maternal health is highlighted; the environmental conditions that hinder the reduction of infant mortality, such as access to drinking water are studied; and finally, the possible strategies to combat non-communicable diseases (NCDs), not included in the Millennium Development Goals are developed.

The last part of the book is bound to set some goals that should be prioritized in African societies in the post 2015 scenario. These include increasing women's participation in politics, generating democratic leadership to stay away from the authoritarian traditions and eliminating corruption in state institutions. Another central theme developed is that of the continent´s relationships with such international actors of great importance in recent decades, as China and other emerging economies, which pose significant potential but also some undesirable effects on development, such as negative environmental impacts and enclave economies unrelated to other productive activities.

In short, considering the development in the current global context is a major challenge, especially in African societies, which present great cultural complexity. This book contributes to this goal, because it presents many variables to consider when thinking, developing and implementing policies and programs with the aim of improving the living conditions of the population, respecting their own cultural identities and attempting to build a relationship which is friendly to the environment in which society develops.

